# Selective Targeting of Epigenetic Readers and Histone Deacetylases in Autoimmune and Inflammatory Diseases: Recent Advances and Future Perspectives

**DOI:** 10.3390/jpm11050336

**Published:** 2021-04-23

**Authors:** Mohammed Ghiboub, Ahmed M. I. Elfiky, Menno P. J. de Winther, Nicola R. Harker, David F. Tough, Wouter J. de Jonge

**Affiliations:** 1 Tytgat Institute for Liver and Intestinal Research, Amsterdam Gastroenterology Endocrinology Metabolism Research Institute, Amsterdam University Medical Centers, University of Amsterdam, 1105 BK Amsterdam, The Netherlands; m.ghiboub@amsterdamumc.nl (M.G.); a.m.elfiky@amsterdamumc.nl (A.M.I.E.); 2 Adaptive Immunity Research Unit, Medicines Research Centre, GlaxoSmithKline, Stevenage SG1 2NY, UK; nicola.r.harker@gsk.com (N.R.H.); david.f.tough@gsk.com (D.F.T.); 3 Department of Medical Biochemistry, Amsterdam University Medical Centers, University of Amsterdam, 1105 AZ Amsterdam, The Netherlands; m.dewinther@amsterdamumc.nl; 4 Department of Medicine, Institute for Cardiovascular Prevention (IPEK), 80336 Munich, Germany; 5 Department of Surgery, University of Bonn, 53127 Bonn, Germany

**Keywords:** epigenetics, histone deacetylases, bromodomain, inhibitor, esterase sensitive motif, autoimmune and inflammatory diseases

## Abstract

Histone deacetylases (HDACs) and bromodomain-containing proteins (BCPs) play a key role in chromatin remodeling. Based on their ability to regulate inducible gene expression in the context of inflammation and cancer, HDACs and BCPs have been the focus of drug discovery efforts, and numerous small-molecule inhibitors have been developed. However, dose-limiting toxicities of the first generation of inhibitors, which typically target multiple HDACs or BCPs, have limited translation to the clinic. Over the last decade, an increasing effort has been dedicated to designing class-, isoform-, or domain-specific HDAC or BCP inhibitors, as well as developing strategies for cell-specific targeted drug delivery. Selective inhibition of the epigenetic modulators is helping to elucidate the functions of individual epigenetic proteins and has the potential to yield better and safer therapeutic strategies. In accordance with this idea, several in vitro and in vivo studies have reported the ability of more selective HDAC/BCP inhibitors to recapitulate the beneficial effects of pan-inhibitors with less unwanted adverse events. In this review, we summarize the most recent advances with these strategies, discussing advantages and limitations of these approaches as well as some therapeutic perspectives, focusing on autoimmune and inflammatory diseases.

## 1. Introduction

The human immune system requires complex mechanisms of regulation to avoid the induction of inappropriate responses, and defects in this regulation result in inflammatory and autoimmune diseases [1,2]. Epigenetics refers to mechanisms that modify cellular and organismal phenotypes without altering the DNA sequence and are linked to modified patterns of gene expression [3]. The critical role of epigenetic mechanisms in regulating gene expression in the immune system is well established, and the dysregulation of epigenetic control contributes to the development of a variety of human diseases [4,5]. Three main mechanisms are commonly ascribed to contribute to epigenetic regulation: (1) RNA interference via noncoding RNAs that modify mRNA translation; (2) DNA methylation, and (3) histone post-translational modification [6,7,8]. All of them can modify the structure of chromatin—the ordered structure of DNA and histones—and ultimately the accessibility of DNA to the transcriptional machinery [6,7,8].

**Figure 1:**
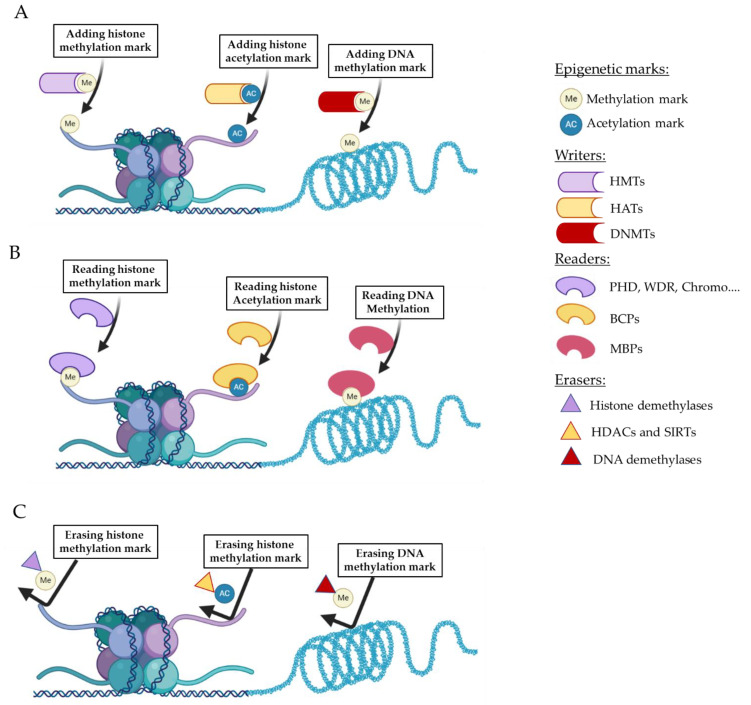
**DNA methylation and histone modifications.** Post-translational modifications of histones and DNA methylation provide a fine-tuned mechanism for regulating chromatin structure and dynamics. Panel (**A**) depicts methylation and acetylation of histone tails that involve the addition of methyl group (Me) and acetyl group (AC), respectively. These processes are catalyzed by the epigenetic writers histone methyltransferases (HMTs) and histone acetylases (HATs), respectively. DNA methylation is catalyzed by the epigenetic writers DNA methyltransferases (DNMTs), including DNMT1, DNMT3a, and DNMT3b, which add a methyl group on to the 5-carbon of the cytosine ring. Panel (**B**) illustrates examples of epigenetic readers that possess specialized domains that recognize specific covalent histone or DNA modifications and respond to upstream signals. Such crosstalk generates a different binding platform for the recruitment of other regulatory proteins, ultimately controlling the chromatin accessibility to transcription factors and gene transcription, such as plant homeodomain (PHD), WD40-repeat (WDR) proteins, and chromo domains that recognize histone methylation marks, broomodomian-containing protiens (BCPs) that recognize histone acetylation marks, and the methyl-CpG-binding proteins (MBPs) that recognize methylation of DNA. Panel (**C**) shows examples of epigenetic eraser proteins that can remove modifications from DNA or histones to regulate gene expression.

Histones are modified in a dynamic way by enzymes that add or erase a wide range of post-translational modifications, including acetyl or methyl groups, to a variety of different amino acids [9]. These modifications can directly affect the interaction between histones and DNA—for example, by changing the charge of the histone—and can also serve as recognition marks for epigenetic “reader” proteins; both of these processes alter DNA accessibility to transcription factors (TFs) (Figure 1) [9]. Enzymes that add or remove histone marks have been referred to as epigenetic “writers” or “erasers”, respectively [9]. Among the wide diversity of possible epigenetic targets, this review will focus on histone deacetylase (HDAC) enzymes and bromodomain (BD)-containing proteins (BCPs), remodelers of histone tails, which play a central role in regulating inducible gene expression involved in immune response [10,11]. HDAC enzymes are examples of epigenetic erasers that remove an acetyl group from histone tails, which then limits the accessibility to DNA at these sites [12]. BCPs are a large family of epigenetic readers that can bind acetylated histones to facilitate recruitment and interactions of TFs [13]. The most extensively studied families of BCPs are BD and extra-terminal domain (BET) proteins, consisting of four individual proteins: BRD2, BRD3, BRD4, and BRDT [14].

Numerous small-molecule inhibitors targeting these proteins have been developed based on preclinical work, suggesting the potential to achieve therapeutic benefit in various human disorders [15,16]. Because of their reported strong antiproliferative and antioncogenic properties, several inhibitors of these epigenetic targets have been investigated in cancer clinical trials, with some HDAC inhibitors FDA approved for certain malignancies [17]. In addition, HDAC inhibitors (HDACi) and BET protein inhibitors (I-BET) have shown strong efficacy in preclinical models of several inflammatory and autoimmune diseases, such as models of inflammatory bowel disease (IBD) and rheumatoid arthritis (RA) [18,19,20,21,22,23,24,25,26]. Although several epigenetic inhibitors are being investigated in human trials, relatively few have progressed into clinical practice and thus far exclusively in the cancer field, largely due to the toxicity profile of these compounds [17,27]. Since these first-generation inhibitors typically target multiple members of the HDAC or BET family, unwanted effects may be linked to a broad impact on transcriptional activity that extends to off-target pathways [28,29].

Recently, increasing effort has been dedicated to developing inhibitors that can achieve a higher degree of selectivity in targeting the epigenetic modulators, which may alleviate safety issues that hold back their translation into clinical use [30,31]. Different strategies are being adopted to tackle this challenge: (1) designing isoform-specific or domain-specific inhibitors that can target only single proteins or individual domains in multidomain proteins; (2) developing a targeted approach that can selectively deliver the drug to the relevant proinflammatory cell types that fuel the inflammation in the disease of interest [32]. In this review, we gather the most recent updates regarding these different strategies and we discuss their potential in paving the way towards the next wave of epigenetic drugs, focusing on inflammatory and autoimmune diseases.

## 2. Methods

To give an overview of existing selective small-molecule inhibitors targeting HDACs and BCPs and gather the most relevant advances in this direction with focus on their use in inflammatory and autoimmune disease models, we performed a literature review in Medline (PubMed) using “HDAC”, “bromodomain”,” inhibitor”, “selective”, “isoform”, “class”, ‘’ esterase sensitive motif’’ ‘’autoimmune diseases’’, and ‘’inflammatory diseases’’ as keywords. Reference lists of existing (systematic) reviews of this topic were searched for additional relevant literature. All included articles were in English. There were no specific inclusion or exclusion criteria for this review. The most referenced articles were selected and are described in the review, with an overview of study characteristics and results shown in Table 1. The figure illustrations were created in BioRender.com.

## 3. Advances in HDAC Selective Targeting in Autoimmune and Inflammatory Diseases

**Figure 2:**
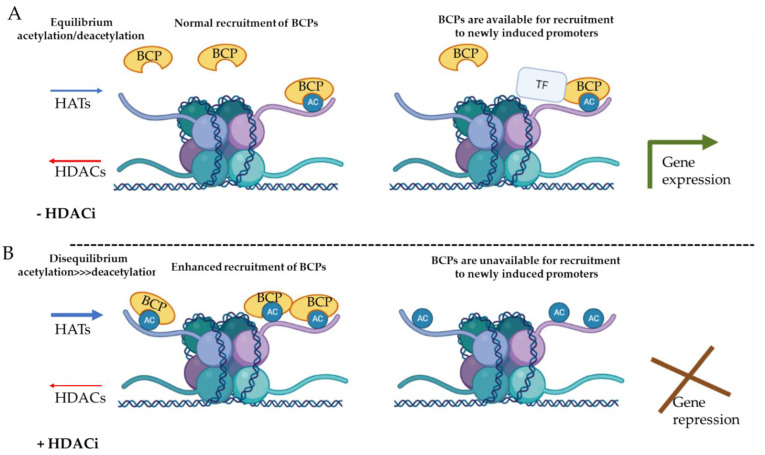
**Possible mechanism for inhibition of inflammatory gene expression by HDACi.** (**A**) In steady state, there is an equilibrium between acetylation (AC) and deacetylation, which maintains a pool of epigenetic readers available for recruitment to newly induced promoters, recruitment of transcription factors (TF), and induction of gene expression. (**B**) Histone deacetylase (HDACs) inhibition increases acetylation of histones that sequester the epigenetic readers, such the bromodomain-containing proteins (BCPs), preventing their recruitment to newly induced promoters.

In humans, the HDAC enzymes family comprises 18 members divided into 4 classes: class I HDACs (HDACs 1–3 and 8), class IIa HDACs (HDACs 4, 5, 7, and 9), class IIb HDACs (HDACs 6 and 10), class III sirtuins (Sirt1–7), and class IV HDACs (HDAC11) [12,33,34]. While histone acetyltransferases (HAT) add acetyl groups to lysine residues, thereby permitting TFs binding and subsequent gene expression, histone deacetylases (HDACs) erase histone acetyl residues, leading to chromatin compaction, generally resulting in gene repression [33,34]. However, in contrast to the common role of HDACs for gene repression, treatment with HDAC inhibitors typically leads to a reduction rather than increase in proinflammatory gene expression by immune cells [35]. This may be due to global histone hyperacetylation induced by HDACi, which results in over-recruitment of epigenetic readers (in steady state, there is an equilibrium between acetylation and deacetylation) [35]. As a consequence, a large pool of epigenetic readers will be sequestrated nonspecifically, reducing the availability of readers for recruitment to newly induced promoters. This subsequently limits the binding of TFs and gene expression induction at these sites, rendering cells less responsive to external inflammatory stimuli [35], as described in Figure 2. HDAC inhibition was also suggested to reduce cytokine expression by promoting mRNA decay. For instance, Grabiec et al. have shown that pan-HDACi can disrupt IL-6 production in RA fibroblast-like synoviocytes (FLS) by accelerating its mRNA breakdown [36].

HDAC enzymes are key regulators of diverse cellular functions, including the inflammatory response, and their dysregulation has been strongly associated with multiple inflammatory diseases [37]. Current research aims to dissect the biological functions of each individual HDAC with attempts to develop class- or isoform-selective HDACi that can maintain similar anti-inflammatory potency of pan-HDACi while providing a better safety profile [38]. A summary of some of the main selective class or isoform inhibitors of HDACs that have been tested in ex vivo and in vivo animal models of inflammatory and autoimmune diseases is provided in Table 1. An approach for drug delivery to specific cell types has also been developed to limit the off-target activity associated with pan-HDACi.

### 3.1. Class-Specific HDACi

#### 3.1.1. Class I-Specific HDACi

Entinostat (MS-275) [39] and Tacedinaline (CI994) [40] are the first potent selective inhibitors of class I HDACs and have shown therapeutic potential by ameliorating inflammation in preclinical models of various inflammatory and autoimmune diseases, including RA [41], Chronic Obstructive Pulmonary Disease (COPD) [42], pancreatitis (19), inflammation associated with angiotensin II-induced hypertension [43], liver fibrosis [44], and lipopolysaccharide-induced acute kidney injury (LPS-AKI) [45]. These beneficial preclinical outcomes were accompanied by a marked reduction in multiple proinflammatory cytokines and leukocyte infiltration [46]. Entinostat treatment reduced cytokine production and suppressed osteoclastic bone resorption in vitro (osteoclast generated from human monocytes), suggesting therapeutic potential in RA and periodontitis [41]. This was borne out by the efficacy of Entinostat in collagen antibody-induced arthritis model where it strongly reduced inflammatory cells infiltration and improved disease score [46]. Notably, Entinostat showed a superior clinical efficacy to pan-HDACi (SAHA) in this model [46]. Similarly, Entinostat was able to significantly affect expression of proinflammatory cytokines in precision-cut lung slices and robustly attenuated inflammatory expression of CXCL1 and neutrophil influx in the lungs in an in vivo mice model of smoking-induced airway inflammation, while the pan-HDACi (SAHA) was without effect in this model [42].

Loh et al. also provided evidence for the importance of class I HDAC inhibition, showing that Entinostat, but not inhibitors of class IIa (PG100) and IIb HDAC (PG50) enzymes, potently suppressed chronic hepatic inflammation and fibrosis in mice [44]. Entinostat has been shown to reduce CD68^+^ macrophage infiltration into aortic tissue in an angiotensin II-induced hypertension murine model [43]. In Cerulein-induced acute and chronic pancreatitis, Entinostat reduced the infiltration of inflammatory immune cells, including macrophages and T cells, and directly disrupted macrophage activation [47]. In terms of safety profiles, although no human clinical data are available yet for inflammatory and autoimmune diseases, the class I HDACi Reminostat exhibited an improved safety profile over pan-HDACi in clinical trials in cancer patients showing no cardiac-related toxicities [48,49]. Two class I HDACis; Etinostat and Mocetinostat, initially exhibited cardiac-related events in early studies. However, further evaluation found that the cardiac events were not related to Mocetinostat [50], while for Entinostat cardiac events were attributed to disparities in drug pharmacokinetics compared with preclinical models, which was mitigated upon redesigning the treatment regimens [51,52]. However, a recent trial for metastatic urethral cancer reported pericardial effusion for one patient that was believed to be Mocetinostat-related [53]. Thus, while class I HDACi appears to have fewer side effects than pan-HDACi, adverse events are still apparent, although some caution should be used in interpreting these events in patients with advanced cancer.

#### 3.1.2. Class II-Specific HDACi

TMP195 (TFMO 2) is a selective, first-in-class, class IIa HDAC inhibitor reported by Lobera et al. [54]. TMP195 exhibited a potent effect on monocyte and macrophage activation in vitro, reducing CCL2 protein secretion and increasing the production of CCL1 by monocyte-derived macrophages and modifying human monocyte responses to the colony-stimulating factors CSF-1 and CSF-2 in vitro [55]. In an LPS acute injury in vivo model, TMP195 inhibited multiple proinflammatory cytokines/chemokines and accumulation of inflammatory cells in the injured kidney [56]. The reno-protective effects of TMP195 observed in this model suggest that targeting class IIa HDACs might be a novel therapeutic strategy for treating renal inflammation, although further investigation of this hypothesis is required.

### 3.2. Isoform-Specific HDACi

Genetic depletion of individual HDACs has demonstrated that these proteins mediate specific and unique functions [57], suggesting therapeutic relevance for selective isoform targeting. While achieving this is a challenge due to conserved structural similarity between HDAC isoforms [12], HDACi has been reported with selectivity for HDAC1, HDAC2, HDAC3 [33,58,59,60,61], HDAC8 [62,63], HDAC6 [64], HDAC11 [65], SIRT1, and SIRT2 [66,67]. However, caution should be taken in interpreting the specificity of the effects of these published inhibitors given the variability of available HDAC assays and the residual dose-dependent effects on other isoforms (Table 1).

#### 3.2.1. HDAC3 Inhibitors

Inhibitors with a high degree of reported selectivity toward HDAC3, including RGFP966, MI192, and ITF3100, have been shown to efficiently attenuate inflammatory responses [33,58,59,68] and to restore LPS tolerance in inflammatory macrophages in vitro [33]. RGFP966 has demonstrated efficacy in preclinical models of diabetes [69], osteoarthritis (OA) [70], and allergy [71] via modulating inflammatory pathways. In diabetic mouse models, RGFP-966 was shown to prevent diabetes-associated liver damage, cerebral ischemia, and cardiomyopathy [72,73,74]. Zhang et al. found that RGFP966 could inhibit the expression of inflammatory markers of OA in rats [70]. Interestingly and unlike pan-HDACi, HDAC3 inhibitor MI192 was able to inhibit the inflammatory response in peripheral blood mononuclear cells (PBMCs) of RA patients but not in PBMCs of healthy control [68]. In line with these observations, inhibition of HDAC3 by the small molecule ITF3100 in RA FLS largely recapitulated the effects of pan-HDACi in suppressing inflammatory gene expression [75]. No effect of HDAC1/2 or HDAC8 inhibition was observed in RA FLS. These data suggest the potential for a clinically relevant advantage of the selective targeting of HDAC3 in RA [75].

#### 3.2.2. HDAC6 Inhibitors

HDAC6 has been extensively studied in various inflammatory settings, and several small-molecule inhibitors have been designed and reported to be selective, such as BML-281 (CAY10603) [76], Ricolinostat (ACY-1215) [77], CKD-506 [78], Tubastatin A [79], and ACY-738 [80]. HDAC6 inhibition has shown efficiency in multiple preclinical models of inflammatory and autoimmune diseases, including IBD [81,82,83], RA [84,85], systemic lupus erythematosus (SLE) [78,86], multiple sclerosis [87], lung inflammatory diseases [88,89], allograft rejection [90], skin inflammatory diseases [91], sepsis [92], and acute liver injury [93]. HDAC6 inhibition has been reported to control immune cell recruitment and to modulate T and B cell differentiation [78,83,94]. In a DSS colitis model, CD19^+^ B cell influx into the inflamed colon was reduced in mice treated with BML-281 [83]. In addition, CKD-506 inhibits NF-κB signaling in intestinal epithelial cells and macrophages and ameliorates murine colitis [81]. LTB2 treatment significantly alleviated DSS-induced colitis in mice [82]. Similarly, in a preclinical murine model of SLE, ACY-738 and CKD-506 were able to modulate both B cell and T cell differentiation, restoring aberrant B cell development and enhancing the frequency of splenic Tregs [78,94]. In addition, in this model, HDAC6 inhibition significantly reduced inflammatory cytokines such as IL-17 and TNF-α and increased TGF-β in serum [78,94]. In experimental autoimmune encephalomyelitis (EAE), ACY-738 delayed disease onset and reduced disease severity [87]. BML-281 blocks inflammatory signaling and caspase-1 activation in the LPS-induced acute lung injury mice model [89].

HDAC6 inhibition impairs effector CD8 T-cell functions during skin inflammation using murine CD8 T cell-related skin disease models, including contact hypersensitivity (CHS) and experimental graft-versus-host disease (GVHD)-like disease [91]. ACY-1215 prevented the development of CHS and GVHD-like disease in vivo by modulating CD8 T cell activation and functions, abrogating the induction of effector T cells from naive CD8 T cells [91]. Tubastatin A downregulated Th17 cell function and suppressed acute lung allograft rejection via the HIF-1α/RORγt pathway in mice [95]. Notably, this effect was observed only with HDAC6 inhibition but not in HDAC1i-, HDAC3i-, HDAC4i-, and HDAC8i-treated recipients [95]. In a murine model of RA, CKD-506 suppressed monocyte/macrophage inflammatory responses, improved Treg function, and ameliorated arthritis severity [84]. Similarly, Tubastatin A showed significant inhibition of IL-6 in paw tissues of arthritic mice in a collagen-induced arthritis model [96].

Although there are no human clinical studies as yet in inflammatory and autoimmune disease patients, similarly to class I HDACi in patients with cancer, HDAC6 inhibitors exhibit an improved safety profile compared with pan-HDACi [50]. For instance, Ricolinostat has shown no drug-related cardiac events in two clinical trial conducted for multiple myeloma patients either alone or in combination with other drugs [97,98]. Life-threatening cardiac arrhythmias are one of the most limiting factors for the use of pan-HDACi in clinical trials [50]. Pan-HDACi is thought to exert this cardiotoxic effect via inhibition of hERG ion channels either directly [99] or indirectly mediated by transcriptional changes that affect ion channel trafficking [100]. Interestingly selective inhibition of HDAC6 was found to stabilize hERG channel expression, which suggests an application for HDAC6 inhibition in long QT syndrome type 2 treatment [101]. To date, seven registered clinical trials are running for other HDAC6 inhibitors, which will help us to better characterize the safety profile of HDAC6 inhibition.

#### 3.2.3. HDAC8 Inhibitors

Some inhibitors of HDAC8 that have been recently reported have shown a marked anti-inflammatory potential in some preclinical models of inflammatory and autoimmune diseases such as sepsis [63], neuro-inflammation [102], and asthma [100]. WK2-16 [102] and PCI-34051 [103] are reported to be the most selective HDAC8 inhibitors. WK2-16 reduced IL-6, TNF-α, and MPP8 expression in both sepsis and LPS-induced neuro-inflammation murine models [63,102] via inhibition of STAT-1/-3 and Akt activation in the absence of an effect on NF-κB or MAPK signaling pathways [63,102]. PCI-34051 was reported to alleviate airway inflammation in a preclinical model of asthma by disrupting HDAC8 interaction with Galectin-3, a protein involved in inflammation and pathogenesis of asthma [104].

#### 3.2.4. Other Isoform-Specific Inhibitors

Santacruzamate A (CAY10683) is a potent HDAC2 inhibitor, with >3600-fold selectivity over other HDACs [105]. Fang-Zhou et al. have demonstrated that Santacruzamate A could suppress neuro-inflammatory responses and TLR4/NF-κB signaling pathways in an animal model of LPS-induced neuro-inflammation [106]. Other HDAC isoforms are reported to regulate the inflammatory response, including HDAC5 [107] and HDAC10 [108], but as of now there are no specific inhibitors to these isoforms. SIS17 is described as a highly selective HDAC11 inhibitor; however, no data are available on studies with this molecule in preclinical models [109].

Finally, selective inhibitors have been developed for some members of the SIRT family (HDAC class III), including EX-527 and AK 7 that target SIRT1 and SIRT2, respectively [66,67]. Although SIRT1 and SIRT2 are implicated in several inflammatory diseases such as RA and IBD, no data are reported for the use of EX-527 and AK 7 in vitro or in vivo models of inflammation.

### 3.3. Cell-Specific Targeted Drug Delivery of Pan-HDACi

Mononuclear myeloid cells play a key role in the pathogenesis of multiple inflammatory diseases but are also critically required for tissue homeostasis and healing [110,111]. Because of the anti-inflammatory activity of HDACi in monocytes/macrophages, selective targeting of these cells could represent an attractive approach for retaining efficacy while minimizing adverse events linked to HDACi in other cell types. A strategy to do so has been developed based on the expression pattern of carboxylesterase 1 (CES1) enzyme (also known as serine esterase 1), which in humans is predominantly expressed in hepatocytes and cells of the mononuclear myeloid lineage, such as monocytes and macrophages, with very little expression reported outside these two sources, mainly in adipose tissue, kidney, and heart [112,113]. CES1 plays a key role in hydrolyzing ester- and amide-bond-containing xenobiotics and drugs [114].

**Figure 3:**
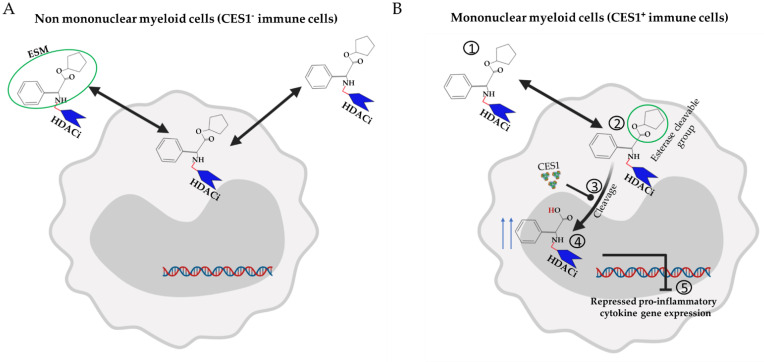
**Selective targeting of myeloid cells with HDACi.** CES-1 expression in humans is restricted to hepatocytes and cells of the mononuclear myeloid lineage, such as monocytes and macrophages. (**A**) When ESM-HDACi enters into CES-1-negative cells such as T and B cells, the compound freely diffuses out of the cells; lack of retention of the HDACi minimizes the pharmacological effect. (**B**) After entry of ESM-HDACi into CES-1-positive cells ①, esters are selectively hydrolyzed by the CES-1 enzyme ②, generating charged acids ③ that are less able to cross the membrane. This leads to intracellular retention of HDACi ④ and enhanced pharmacological effect (e.g., histone hyperacetylation and repression of proinflammatory genes) ⑤.

Esterase sensitive motif (ESM) technology has been employed to selectively target CES1-expressing cells. Small-molecule inhibitors are tagged with the ESM motif, the ESM-tagged inhibitors enter cells, and when CES1 is expressed, the ESM motif is hydrolyzed into an acid [32] as described in Figure 3. In acid form, the compound is less able to cross the plasma membrane, thus increasing retention and therefore potency in CES1 expressing cells [32] (Figure 3). Such targeted molecules have shown efficacy in a preclinical model of RA in which transgenic mice that express human CES1 under the CD68 promotor were generated to allow human CES1 expression in mononuclear myeloid cells [32]. ESM-HDACi achieved clinical improvement at doses as low as 1 mg/kg compared with 100 mg/kg of conventional pan-HDACi (SAHA) needed to achieve a similar clinical response [32]. ESM-HDACi was tolerated up to 30 mg/kg in vivo dosing [32].

In a phase 1 clinical study, ESM-HDACi proved to be safe and well tolerated while showing efficient and sustainable accumulation in blood monocytes [115]. This sparked the interest to further explore this strategy in other inflammatory disease models. In both acute DSS colitis [116] and acute peritonitis [117] models, ESM-HDACi impaired the differentiation of monocyte in inflamed tissue, which translated into modestly improved colitis [116]. Exploring this strategy in a variety of inflammatory disease models may identify the best application of this approach given the complex role of these cells in mediating the inflammatory response in different diseases [118,119]. In addition, using ESM technology with more selective HDAC inhibitors (class or isoform specific) would provide better therapeutic potential.

## 4. Advances in Selective Targeting of BCPs

**Figure 4:**
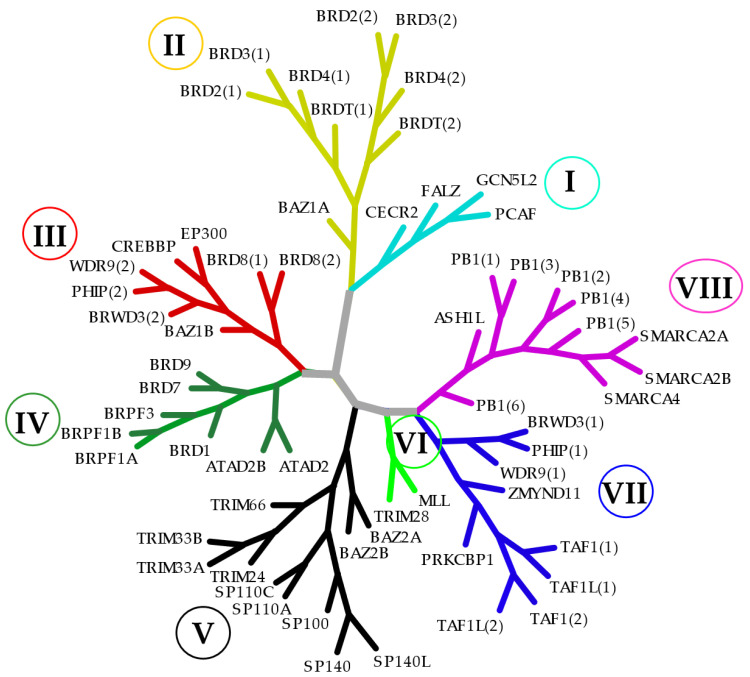
**Phylogenetic tree of the human bromodomain-containing protein subgroups.** On the basis of sequence homology, BCPs are classified into eight different subgroups (families). The distinct families are indicated by Roman numbers (I–VIII) in circles and illustrated with different colors.

BCPs are group of epigenetic readers that recognize acetylated lysine residues on histone tails and play a role in modulating DNA accessibility to TFs and the transcriptional machinery [120]. Disturbance in BCP function has been reported as a key contributor to a large variety of diseases [15]. In humans, if we exclude splice variants, there are around 56 BDs and 42 BCPs characterized [121]. Based on sequence homology, BCPs are classified into eight different subgroups [120], as described in Figure 4. BCPs are tractable to small-molecule antagonists that prevent protein–protein interaction between BCPs and acetylated histones and transcription factors [122]. Although numerous compounds targeting BCPs (primarily BET family BCPs) have displayed promising therapeutic potential in preclinical models of cancer and autoimmunity/inflammation, these compounds have not yet been approved by FDA [123]. The majority of BCP inhibitors lack selectivity for individual BCPs or a specific domain, and as BCPs regulate the expression of a plethora of genes and can be ubiquitously expressed, therapeutic translation into the clinic has been restricted by multiple adverse events [121]. To reduce the breadth of effects observed with first-generation BCP inhibitors, efforts have been made to achieve better selectivity amongst BRDs, as well as to develop specific cell type delivery of small molecules targeting BCPs. Three main advances in this direction are discussed below. A summary of some of the main tested selective domain or isoform inhibitors of BCPs in ex vivo and in vivo animal models of inflammatory and autoimmune diseases are described in Table 1.

**Table 1:** Summary of some of the main tested selective inhibitors of HDACs and BCPs in ex vivo and in vivo models of inflammatory and autoimmune diseases.

Inhibitors	Targets	Degree of Selectivityin Cell Free Assay	Preclinical Models of Inflammatory and Autoimmune Diseases	Key Findings
Entinostat (MS-275) [39]	Class I HDAC	IC50s of 243, 453, and 248 nM for HDAC1, HDAC2, and HDAC3, respectively.	Collagen antibody-induced arthritis (mouse and rat) [46]	While pan-HDACi (SAHA) could not inhibit the onset of arthritis, Entinostat displayed strong antirheumatic activities.
			Cigarette smoke-induced airway inflammation (mouse) [42]	While Entinostat attenuated inflammatory expression and neutrophil influx in the lungs, pan-HDACi (SAHA) was without effect.
			Thioacetamide-induced hepatic inflammation (mouse) [44]	Entinostat but not class IIa and IIb HDACi suppressed chronic hepatic inflammation and fibrosis.
			Cerulein-induced acute and chronic pancreatitis (mouse) [47]	Reduced infiltration of inflammatory immune cells.
Tacedinaline (CI994) [40]	Class I HDAC	IC50s of 0.9, 0.9, 1.2, and >20 μM for human HDAC 1, 2, 3, and 8, respectively	Titanium particle-induced calvarial osteolysis (mouse) [124]	Tacedinaline inhibited osteoclastogenesis through targeting NF-κB and the downstream c-Fos/NFATc1 signaling pathway.
TMP195 (TFMO 2) [54]	Class IIa HDAC	IC50s of 59, 60, 26, and 15 nM for HDAC4, HDAC5, HDAC7, and HDAC9, respectively. 100-fold selectivity over other HDACs (IC50s >10 µM)	Lipopolysaccharide-induced acute kidney injury (mouse) [56]	TMP195 inhibited multiple proinflammatory cytokines/chemokines and reduced the accumulation of inflammatory immune cells in the injured kidney.
RGFP966 [125]	HDAC3	IC50 of 0.08 μM inhibits HDAC3 > 200-fold selectivity over other HDACs	Diabetic models (mouse) [72,73,74]	RGFP966 prevented diabetes-associated liver damage, -cerebral ischemia and –cardiomyopathy.
			Osteoarthritis model (rat) [70]	RGFP966 inhibited the expression of inflammatory markers via modulating HDAC3/NF-kB pathway.
MI192 [68]	HDAC3	IC50s of 16 and 30 nM, for HDAC2 and HDAC3, respectively.	Ex vivo-stimulated human peripheral blood mononuclear cells (PBMCs) of RA patients [68]	Unlike pan-HDACi, MI192 inhibited inflammatory response in PBMC of RA patients but not in PBMCs of healthy control.
Santacruzamate A (CAY10683) [105]	HDAC2	IC50 of 119 pM for HDAC2 and with >3600-fold selectivity over other HDACs.	LPS-induced neuro-inflammation (mouse) [106]	Santacruzamate A suppressed neuro-inflammatory responses and TLR4/NF-κB signaling pathways.
Tubastatin A [79]	HDAC6	IC50 of 15 nM for HDAC6. It is selective against all the other HDACs (1000-fold) except HDAC8 (57-fold).	Orthotopic lung transplantation model (mouse) [95]	Tubastatin A downregulated Th17 cell function and suppressed acute lung allograft rejection.Notably, this effect was observed only with HDAC6 inhibition but not with HDAC1i-, HDAC3i-, HDAC4i-, and HDAC8i-treated mice.
			Collagen-induced arthritis (mice) [96]	Tubastatin A reduced IL-6 in paw tissues of arthritic mice.
ACY-738 [80]	HDAC6	IC50 of 1.7 nM for HDAC6 and 60- to 1500-fold selectivity over class I HDACs	Model of systemic lupus erythematosus (SLE) (mouse) [94]	ACY-738 modulated both B cell and T cell differentiation, restored the aberrant B cell development and enhanced the frequency of splenic Tregs.
			Experimental autoimmune encephalomyelitis model (mouse) [87]	ACY-738 delayed disease onset and reduced disease severity.
BML-281 (CAY10603) [76]	HDAC6	IC50s of 2 pM; for HDAC6. BML-281 also inhibits HDAC1, HDAC2, HDAC3, HDAC8, and HDAC10, with IC50s of 271, 252, 0.42, and 90.7 nM.	DSS-induced colitis (mouse) [83]	CD19^+^ B cell influx into inflamed colon was reduced in mice treated with BML-281.
			LPS-induced acute lung injury (mouse) [89]	BML-281 blocks inflammatory signaling and caspase-1 activation.
LTB2 [82]	HDAC6	IC50 value of 3.9 nM	DSS-induced colitis (mouse) [82]	LTB2 prevented DSS-induced colitis.
Ricolinostat (ACY-1215) [77]	HDAC6	IC50 of 5 nM for HDAC6. Ricolinostat also inhibits HDAC1, HDAC2, and HDAC3 with IC50s of 58, 48, and 51 nM, respectively.	Contact hypersensitivity (CHS) and experimental graft-versus-host disease (GVHD)-like disease (mouse) [91]	Ricolinostat prevented the development of CHS and GVHD-like disease by modulating CD8 T cell activation and functions; abrogated the induction of effector T cells from naive CD8 T cells
CKD-506 [78]	HDAC6	IC50 of around 5 nM. IC50 values for HDAC1, HDAC2, HDAC7, and HDAC8 were in the range of 2000–5000 nM.	DSS- and adoptive T cell transfer-induced colitis (mouse) [81].	CKD-506 ameliorated weight loss, disease activity, and histopathologic score and downregulated proinflammatory cytokines production.
			Model of SLE (mouse) [78]	CKD-506 modulate both B cell and T cell differentiation, restoring the aberrant B cell development and enhancing frequency of splenic Tregs
			Adjuvant-induced arthritis (mouse) [84]	Suppresses monocytes/macrophages inflammatory responses, improves Treg function, and ameliorates arthritis severity.
WK2-16 [102]	HDAC8	-	Sepsis and LPS-induced neuro-inflammation (mouse) [63,102]	WK2-16 was able to reduce IL-6, TNF-α and MPP8.
PCI-34051 [103]	HDAC8	IC50 of 10 nM for HDAC8 and with 200-fold selectivity over HDAC1 and 6 and more than 1000-fold selectivity over HDAC2, 3, and 10.	Ovalbumin-induced asthma (mice) [104]	PCI-34051 alleviated airway inflammation and disrupted HDAC8 interaction with Galectin-3, a protein involved in inflammation and pathogenesis of asthma.
ESM-HDAC528 [32]	Pan-HDAC	Selective accumulation of pan-HDACi in CES1^+^ cells.	Arthritis model [32]	ESM-HDAC528 achieved clinical improvement at lower dose of 1 mg/kg compared with 100 mg/kg of conventional pan-HDACi (SAHA)
			DSS-induced colitis [116]	ESM-HDACi impaired monocytes differentiation in the inflamed tissue, and this was translated into modest improved colitis
ZL0420 and ZL0454 [126,127]	BRD4	For ZL0420, an IC50 of 27 nM against BRD4 BD1 and 32 nM against BRD4 BD2, and for ZL0454, an IC50 of 49 and 32 nM for BD1 and BD2.	TLR3-mediated acute airway inflammation (mice) [126]	The infiltration of neutrophils into the airway fluids and cytokine expression in the lung tissue were more effectively blocked by BRD4 inhibitors than pan-I-BET; (+)-JQ1 or RVX-208
GSK761 [4]	SP140	IC50 value of 77.79 ± 8.27 nM	CD14^+^ macrophages isolated from Crohn’s disease colonic tissues [4]	GSK761 reduced the spontaneous secretion of proinflammatory cytokines by macrophages
I-BRD9 [128,129]	BRD9	pIC50 of 7.3 for BRD9, with pIC50 of 5.3 against BRD4.	Nur77 knockout-induced obesity (mice) [130]	Combining I-BRD9 with calcipotriol regulated the gut microbiota and improved intestinal mucosal barrier function.
GSK046 (iBET-BD2) [131]	BD2 of BET proteins	IC50s of 264 nM (BRD2 BD2), 98 nM (BRD3 BD2), 49 nM (BRD4 BD2), and 214 nM (BRDT BD2).	Models of RA and psoriasis (mouse) [131]	Immunomodulatory effects.
GSK778 (iBET-BD1) [131]	BD1 of BET proteins	IC50s of 75 nM (BRD2 BD1), 41 nM (BRD3 BD1), 41 nM (BRD4 BD1), and 143 nM (BRDT BD1), respectively	Cancer model (mouse) [131]	GSK778 phenocopied the effects of pan-BET inhibitors in cancer models.

### 4.1. Domain-Selective Targeting (BD1 or BD2 Targeting)

The BET protein family of BCPs comprise the ubiquitously expressed BRD2, BRD3, and BRD4 and the testis-restricted BRDT, all of which harbor two highly conserved tandem bromodomains, BD1 and BD2, allowing them to recognize acetylated lysines [131,132]. BET proteins are well recognized as drug targets for multiple human diseases [131]. Most potent and selective BCP inhibitors reported target all eight BDs of the BET family (i.e., BD1 and BD2 in BRD2, 3, 4 and T), and include I-BET762 [133], (+)-JQ1 [134], I-BET151 [122], and I-BET726 [135]. These compounds display similar affinities to BD1 and BD2 [131]. Such compounds have been utilized to demonstrate the function of these proteins in selectively regulating expression of genes with high therapeutic interest for several human inflammatory diseases [21,131,136]. Recent reports studying the molecular mechanisms of BET protein binding demonstrate differential binding of BD1 and BD2 to different targets [121,136]. These mechanistic studies suggest a model where BD1 is essential for BET protein binding to di-acetylated nucleosomes, while BD2 is more relevant to binding to TFs and protamines [121,136]. Therefore, selective inhibition of either BD1 or BD2 binding might promote different functional consequences that allow for more selectivity in functional effect and an improved safety profile to be explored in different diseases settings [121,136].

BD1 and BD2 have high sequence similarity within the acetyl–lysine binding site but exhibit distinct recognition patterns of acetylated histone peptide targets [137]. Accordingly, BD1 and BD2 of BRD4 can both recognize acetylated H4 peptide [137]. However, only BD1 has been shown to specifically recognize N-terminal-acetylated H4 peptides in a sequence-dependent manner, while BD2 is more promiscuous [131]. In addition, BD1 favors binding to di-acetylated residues on histone H4, particularly H4 K5ac/K8ac, whereas BD2 is more permissive and can accommodate a variety of di-acetylated peptides [131]. Importantly, despite the ability of BD2 to bind acetylated histones in vitro, biochemical studies have indicated that BD1 of the BET proteins is mainly responsible for chromatin binding [137,138]. This suggests that selective inhibition of BD1 or BD2 would be expected to yield distinct subsets of effects other than observed with pan-BET inhibitors [139]. Emerging data with domain-selective inhibitors are providing support for this idea.

**Figure 5:**
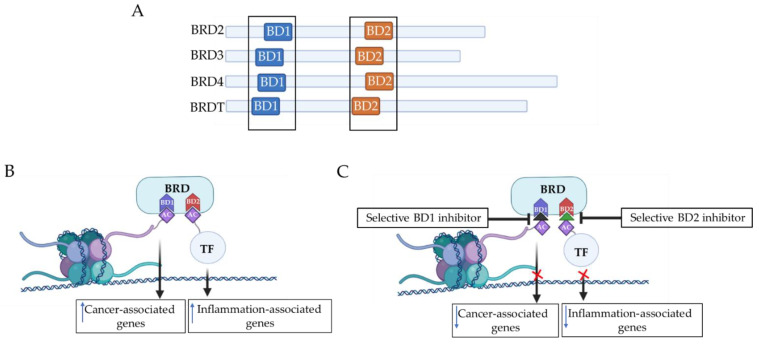
**Selective domain targeting of BD1 and BD2.** (**A**) BET proteins family of BCPs comprise the ubiquitously expressed BRD2, BRD3, and BRD4 and BRDT, which all harbor two highly conserved tandem bromodomains, BD1 and BD2, allowing them to recognize acetylated lysine. (**B**) BD1 and BD2 have distinct functions. BD1 binding is sufficient to maintain homeostatic and oncogenic gene transcription programs, while BD2 binding is required to promote an inflammatory transcriptional response. (**C**) Domain-selective inhibitors for BD1 and BD2 show preferential effects on different types of cellular functions [141].

ABBV-744 is a BET inhibitor that shows preferential inhibition of the BD2 domain [140]. ABBV-744 has demonstrated a robust activity in prostate cancer xenografts and exhibited fewer platelet and gastrointestinal toxicities compared with the dual-bromodomain BET inhibitors (DbBi) [140]. Recently, Gilan et al. described the development of GSK778 (iBET-BD1) and GSK046 (iBET-BD2), the first highly selective small-molecule inhibitors of BET-BD1 and BET-BD2, respectively [131]. This advance has helped to highlight more distinct roles of BD1 and BD2 (Figure 5). iBET-BD1 showed a selectivity of ≥130-fold for BRD4 BD1, and iBET-BD2 showed selectivity of ≥130-fold for BRD4 BD2 [131]. In this study, iBET-BD1 was able to replicate the effects of pan-BET inhibitor I-BET151 in human cancer cell lines, such as inducing cell cycle arrest and clonogenic capacity [131]. Notably, the effects of iBET-BD2 were less pronounced in this setting [131]. This was explained by the ability of iBET-BD1 to reduce the chromatin binding of all BET proteins, including BRD4, which is known to be critical for maintaining oncogenic and homeostatic transcriptional programs, while iBET-BD2 failed to interfere with BRD 2, 3, or 4 binding in an in vitro THP-1 homeostatic model. Interestingly, in the context of inflammatory stimulus-induced in vitro models, iBET-BD2 was able to strongly reduce BRD2 and BRD3 binding while only sparing BRD4 binding and was able to inhibit the IFNγ-induced transcriptional program [131]. Despite no effects on cancer cell proliferation or survival, iBET-BD2, like iBET-BD1, exerted immunomodulatory effects by decreasing the expression of proinflammatory cytokines in in vitro assay [131]. These mechanistic studies of BET BD1/BD2 functionality indicate a model where BD1 binding is sufficient to maintain homeostatic and oncogenic gene transcription programs, while both BD1 and BD2 binding is required to promote an inflammatory transcriptional response (Figure 5). This observation implies that selective inhibition of BD2 could be a promising strategy for treatment of inflammatory diseases with a potential for an improved safety profile. Indeed, iBET-BD2 was shown to provide efficacy in animal models of RA and psoriasis [131]. Overall, these findings of differential functions for BD1 and BD2 (Figure 5) indicate a therapeutic rationale founded on distinct BET BD-selective targeting in cancer and immuno-inflammatory diseases.

### 4.2. Selective Targeting of Single BCP

#### 4.2.1. BRD4 Inhibitors

Important advances in new technologies and tools such as assay kits for screening inhibitors, Targetome, BromoMELT, BromoScan assay, and others, have revolutionized the discovery of series of diverse small molecules that selectively target a single BCP. These include ZL0420 and ZL0454, potent selective inhibitors of BRD4 [126,127]. ZL0420 has reported IC50 values of 27 nM against BRD4 BD1 and 32 nM against BRD4 BD2, while ZL0454 shows IC50 value of 49 and 32 nM for BD1 and BD2, respectively [126,127]. These molecules have been utilized to explore the potential of selective targeting of BRD4 in airway inflammation, based on reports of a critical role for BRD4 in NF-κB-mediated epithelial–mesenchymal transition in an in vitro model of airway epithelial cell culture and in in vivo murine models of pulmonary fibrosis and TLR3-mediated acute airway inflammation [27]. Intranasal administration of poly(I:C) induced a substantial increase of total cells and neutrophils into the airway fluids, and cytokine expression in the lung tissue [126]. These changes were more effectively blocked by BRD4 inhibitors than by pan-I-BET; (+)-JQ1 or RVX-208 [126]. In another study, MS436, a compound that preferentially targets the first bromodomain of BRD4, blocked the transcriptional activity of BRD4 in the NF-κB-directed production of nitric oxide and IL-6 [142]. In addition to the small molecules inhibiting BET BD protein–protein interactions described above, molecules able to degrade BET proteins based on PROTAC technology have been developed. Notably, some of these have been reported to have BET isoform selectivity, such dBET57 [143] and QCA570 [144], which have been reported to selectively degrade BRD4. To date, however, no in vivo studies with these molecules are reported.

#### 4.2.2. SP140 Inhibitor

The development of inhibitors targeting BCPs proteins other than BET in inflammatory and autoimmune diseases are rare, although some advances have been reported. Another BCP with reported therapeutic potentials is speckled 140 KDa (SP140), which belongs to the SP100 family of proteins that also includes SP100, SP110, and SP140 L [4]. Genetic and epigenetic alterations in the SP140 locus have been strongly associated with autoimmune and inflammatory diseases, including Crohn’s disease (CD) and multiple sclerosis [145,146]. SP140 is predominantly expressed in immune cells, suggesting an interesting therapeutic potential [4]. Ghiboub et al. have described the first selective small-molecule inhibitor of SP140 (GSK761). GSK761 was shown to compete with the N-terminal tail of histone H3 for interactions with the SP140 BRD-PHD module [4]. GSK761 decreased the differentiation of monocytes into inflammatory macrophages and LPS-induced inflammatory activation, whilst inducing the generation of CD206^+^ regulatory macrophages that mark anti-TNF remission induction in CD patients. Notably, ex vivo treatment of CD14^+^ macrophages isolated from CD intestinal mucosa with GSK761 inhibited the spontaneous expression of cytokines, including TNF [4]. While this study identifies SP140 as a druggable epigenetic reader and potential therapeutic target for CD, GSK761 however shows poor in vivo pharmacokinetics, potentially restricting its use in vivo [4].

#### 4.2.3. BRD9 Inhibitors

BRD9 is part of the SWI/SNF remodeling BAF complex [147], and selective BRD9 inhibitors, I-BRD9 and BI-7273, have been described [128,129]. Evidence for the importance of BRD9 in immune function has come from studies in the T cell transfer colitis model, where T effector cells were co-transferred with either BRD9-depleted or normal Treg cells [148]. Notably, BRD9-deficient Tregs, unlike control Tregs, failed to prevent colitis development in recipient mice. [148]. As BRD9-deficient Tregs were also defective in the context of tumor immunity, the authors suggested that small-molecule drugs could be useful to fight cancer. Interestingly, a study by Qingqing Lv et al. showed that calcipotriol combined with I-BRD9 can regulate the gut microbiota, improve intestinal mucosal barrier function, and reduce LPS absorption into the blood [130].

#### 4.2.4. CREB Inhibitor

The BCP cyclic AMP response element-binding protein (CREB) is a transcriptional coactivator of many different transcription factors and plays an essential role in regulating the immune response [149], including mediating TNF-α and IL-10 production in macrophages and controlling cytokine expression by Th1 (IL-2 and IFN-γ) and Th2 cells (IL-4 and IL-13) through the regulation of IFN-γ production [149]. Dysregulation of CREB has been associated with several immune-mediated diseases [149,150,151]. For instance, CREB was shown to play a role in synovial cell hyperfunction in patients with RA [151] and intestinal barrier dysfunction in IBD [150]. Thus, targeting CREB selectively may yield therapeutic benefits for inflammatory diseases. PF-CBP1 and KG-501 have been reported as potent selective inhibitors of CREB by Eugene L et al. [152] and Jennifer L et al. [153], respectively. These compounds target specifically the BD in CREB protein [152,153]. PF-CBP1 demonstrated strong potential to reduce proinflammatory cytokines in human macrophages in vitro [152]. Notably, the authors observed several other genes that were affected by PF-CBP1 but not by pan-BET inhibitor, including REL, RELB, CCL2, CCL3, MRC1, and NFKBIA [152]. These data highlight the effects of CBP pharmacological inhibition on specific and distinct molecular targets and suggest that CBP inhibitors could be used to investigate therapeutic opportunities in inflammation that possess a molecular etiology mechanistically different to BET-associated inflammation.

#### 4.2.5. BRPF Inhibitors

BRD and plant homeodomain finger-containing (BRPF) family proteins consist of three members: BRPF1, BRPF2 (BRD1), and BRPF3 [154]. Julia et al. have reported three potent and selective inhibitors: one (PFI-4) with high selectivity for the BRPF1B isoform and two pan-BRPF bromodomain inhibitors (OF-1, NI-57) [155]. Intriguingly, the inhibitors impaired RANKL-induced differentiation of primary murine bone marrow cells and human primary monocytes into bone-resorbing osteoclasts by specifically repressing transcriptional programs required for osteoclastogenesis [155].

### 4.3. Cell-Specific Targeted Drug Delivery of I-BET

As discussed above, the ESM technology can be used to preferentially target small-molecule inhibitors to myeloid cells. GlaxoSmithKline has utilized this approach to generate myeloid-targeted ESM-I-BET compounds [156]. However, no preclinical data are available yet for these compounds [156].

## 5. Conclusions/Perspectives

While progress is being made regarding the development of more isoform/domain-selective HDAC and BCP inhibitors, further pre- and clinical studies of these molecules are required to better characterize their efficacy and safety profiles. Although new HDAC and BCP inhibitors display high affinity towards specific classes, isoforms, or domains, most still retain residual effects on one or more other epigenetic enzymes, as described in the Table 1. Thus, efforts should continue towards developing new isoform/domain-selective inhibitors with improved specificity to avoid potential off-target effects. Meanwhile, two valuable approaches for future studies can help improve safety and guide applications into the inflammatory disease field. The first approach would involve examining individual HDAC and BCP expressions in different inflammatory diseases, as reviewed for IBD here [157]. This may better identify the relevant individual HDAC/BCP for each disease setting and guide selective inhibitor design to target the relevant isoform/domain for each disease. A second approach would involve conducting transcriptional analysis studies that compare the impact of individual isoform/domain knockdowns/inhibitions in different in vitro and in vivo systems. This can help to identify targets that show efficient modulation of inflammatory pathways without impacting other central/homeostatic pathways upon their inhibition. Results from such studies can guide the development and application of further selective HDAC/BCP inhibitors in inflammatory and autoimmune diseases.

## Data Availability

Not applicable.

## Abbreviations

HDAC: histone deacetylase; BD: bromodomain; BRD: BET bromodomain-containing protein; IC50: half-maximal inhibitory concentration; SP140: speckled 140 KDa, CES1: carboxylesterase 1; BCP: Bromodomain containing protein.
